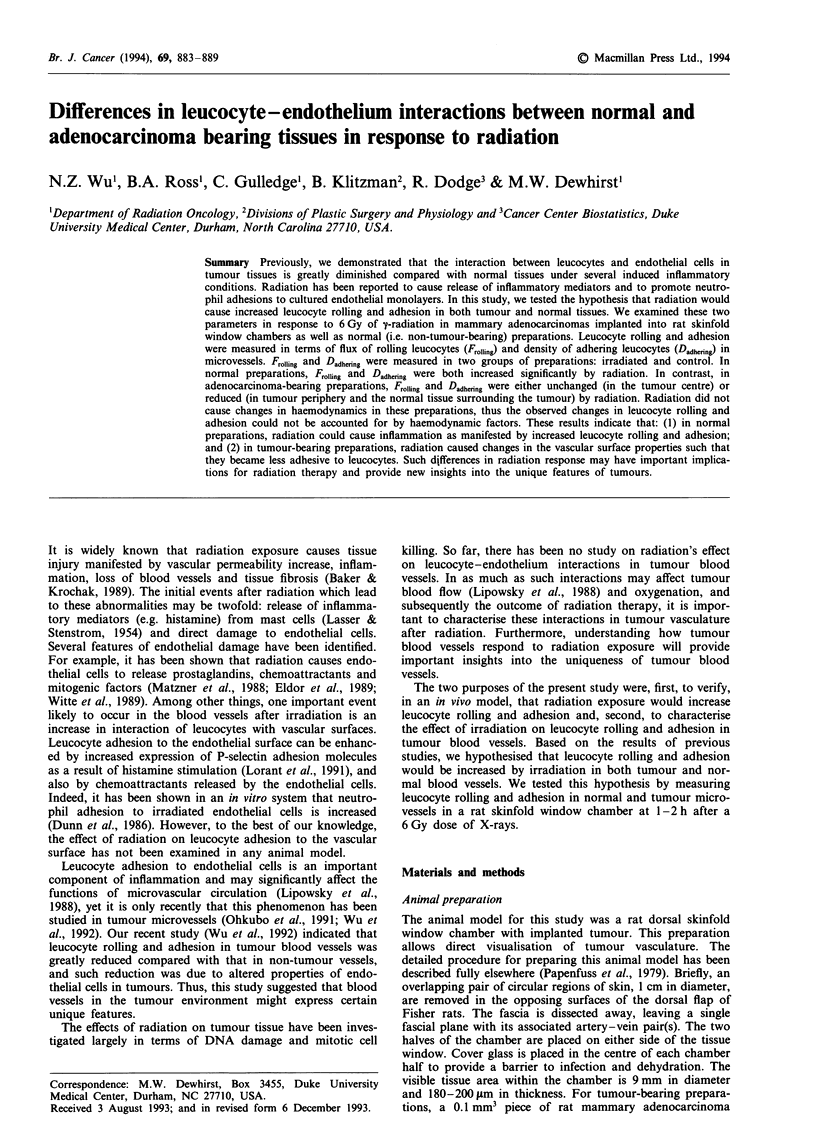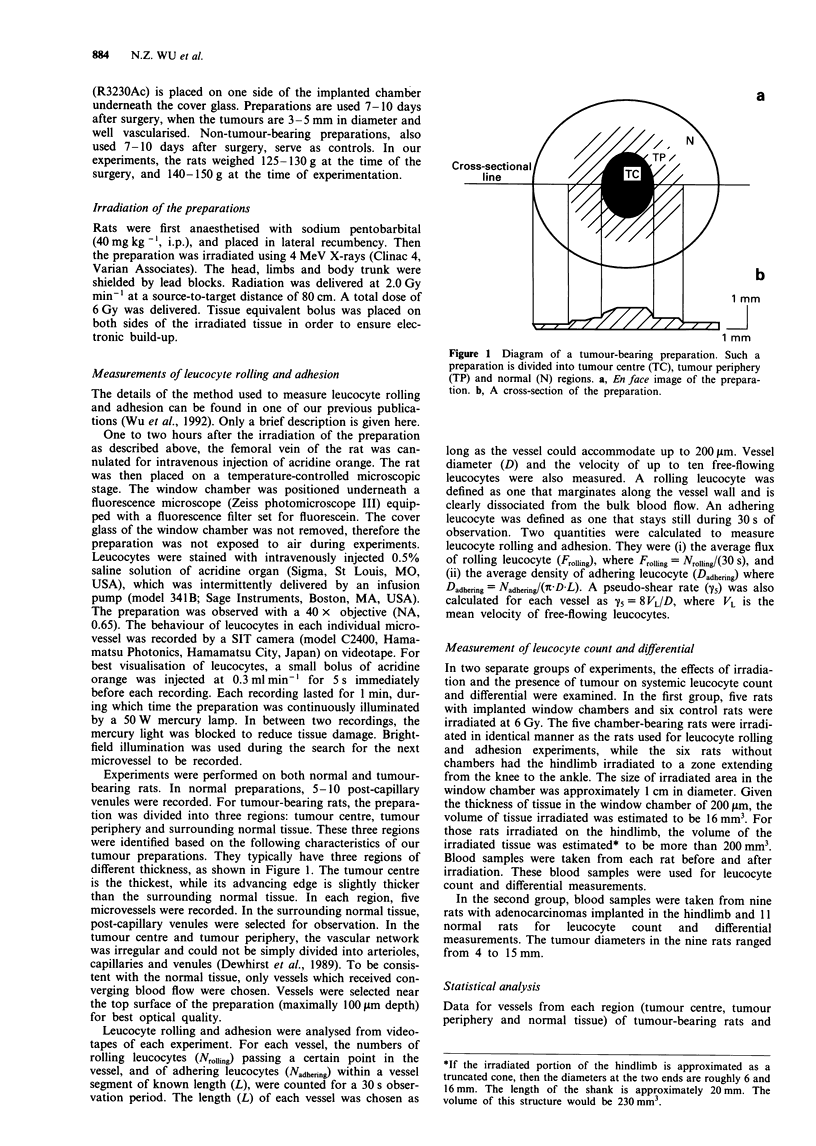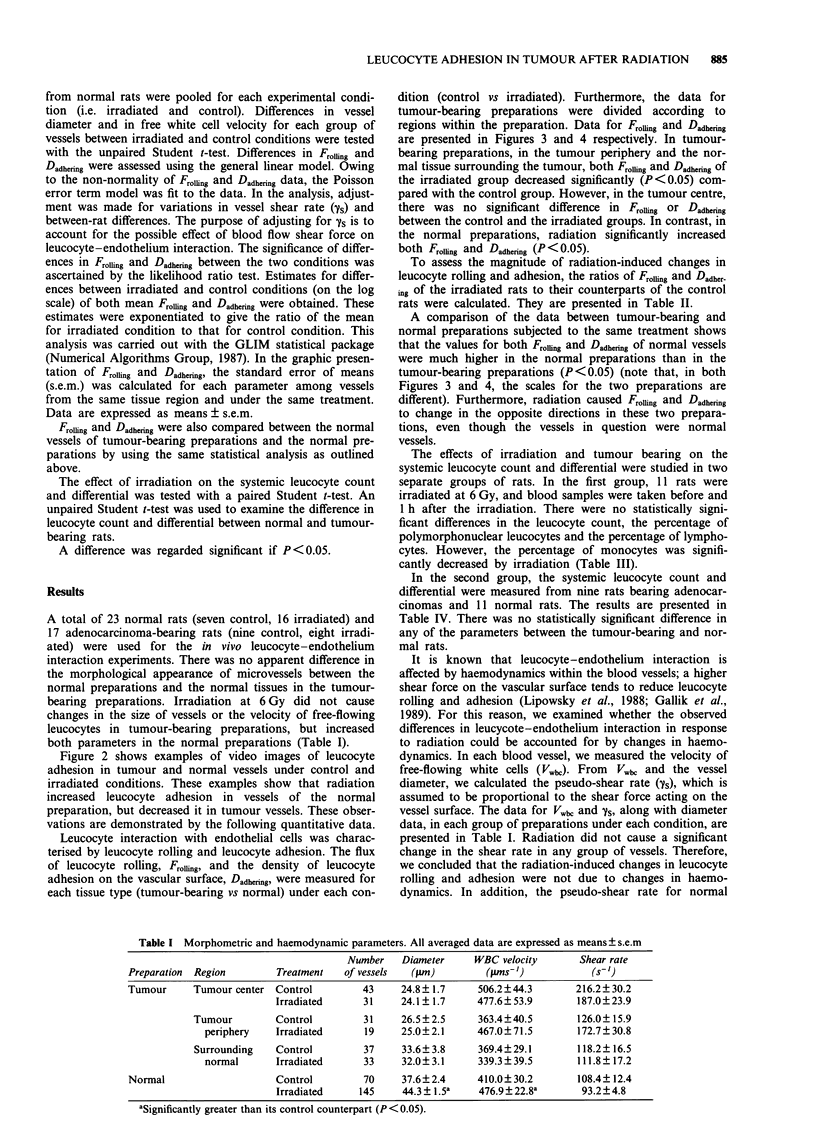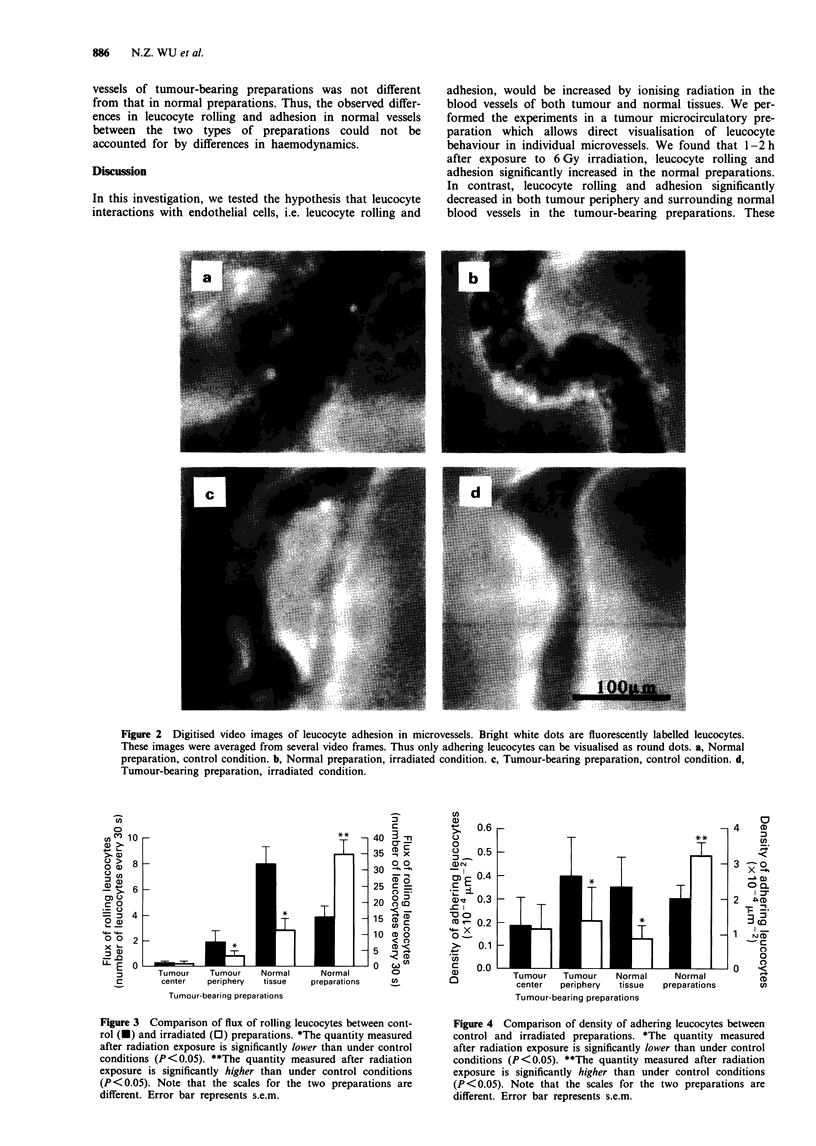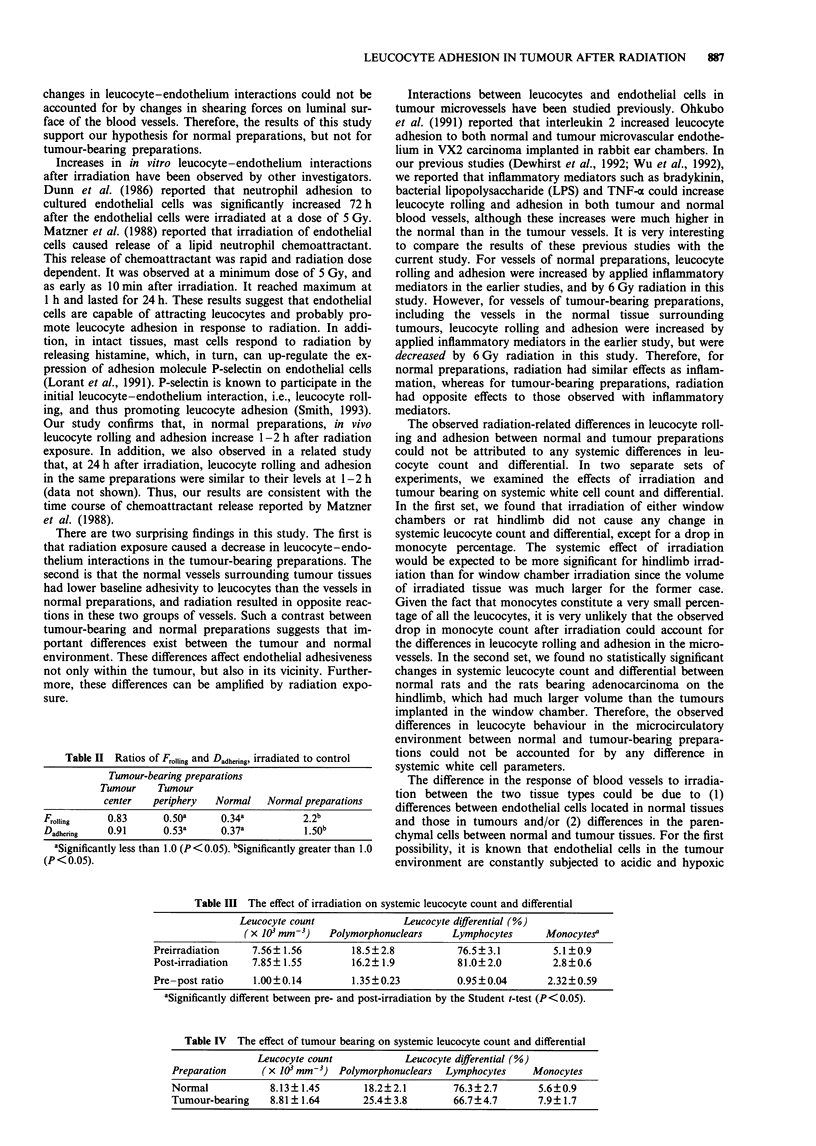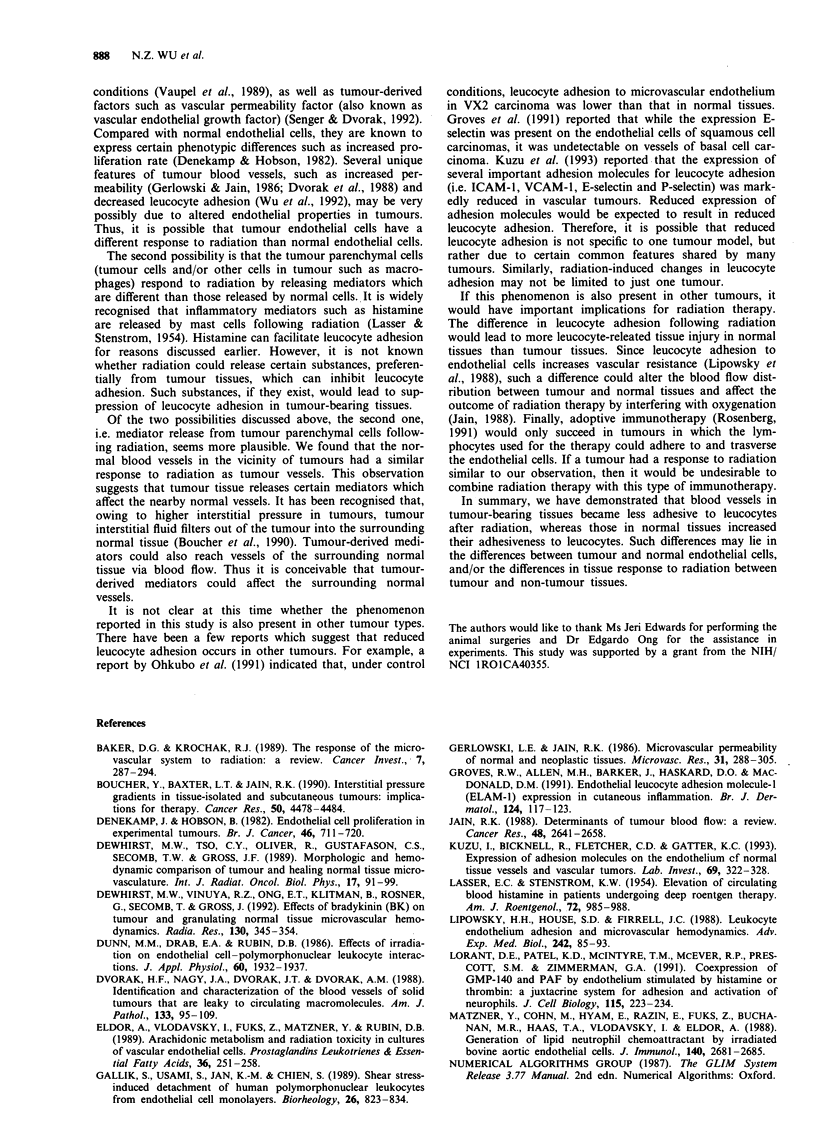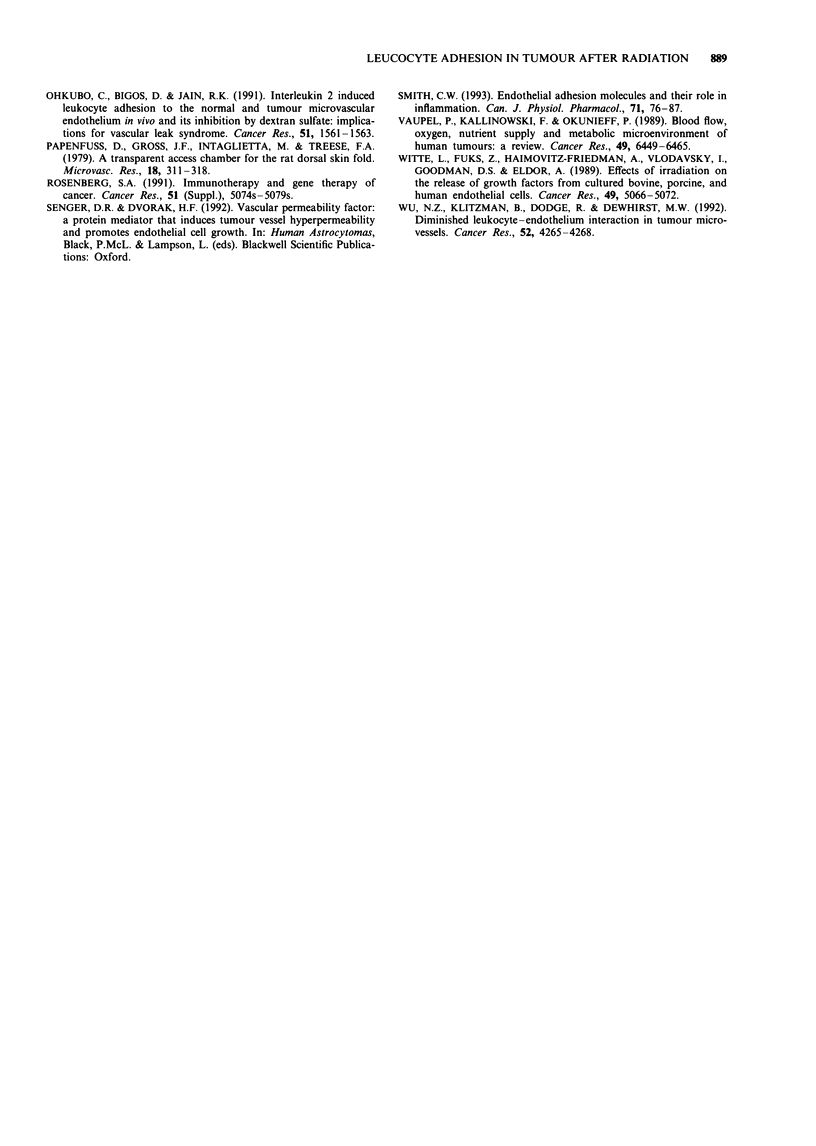# Differences in leucocyte-endothelium interactions between normal and adenocarcinoma bearing tissues in response to radiation.

**DOI:** 10.1038/bjc.1994.171

**Published:** 1994-05

**Authors:** N. Z. Wu, B. A. Ross, C. Gulledge, B. Klitzman, R. Dodge, M. W. Dewhirst

**Affiliations:** Department of Radiation Oncology, Duke University Medical Center, Durham, North Carolina 27710.

## Abstract

**Images:**


Br. J. Cancer (1994), 69, 883-889 © Macmillan Press Ltd., 1994

### Differences in leucocyte-endothelium interactions between normal and adenocarcinoma bearing tissues in response to radiation

N.Z. Wu[1], B.A. Ross[1], C. Gulledge[1], B. Klitzman[2], R. Dodge[3] & M.W. Dewhirst[1]

[1]*Department of Radiation Oncology,* [2]*Divisions of Plastic Surgery and Physiology and* [3]*Cancer Center Biostatistics, Duke University Medical Center, Durham, North Carolina 27710, USA.*

**Summary** Previously, we demonstrated that the interaction between leucocytes and endothelial cells in tumour tissues is greatly diminished compared with normal tissues under several induced inflammatory conditions. Radiation has been reported to cause release of inflammatory mediators and to promote neutrophil adhesions to cultured endothelial monolayers. In this study, we tested the hypothesis that radiation would cause increased leucocyte rolling and adhesion in both tumour and normal tissues. We examined these two parameters in response to 6 Gy of γ-radiation in mammary adenocarcinomas implanted into rat skinfold window chambers as well as normal (i.e. non-tumour-bearing) preparations. Leucocyte rolling and adhesion were measured in terms of flux of rolling leucocytes ($F_{rolling}$) and density of adhering leucocytes ($D_{adhering}$) in microvessels. $F_{rolling}$ and $D_{adhering}$ were measured in two groups of preparations: irradiated and control. In normal preparations, $F_{rolling}$ and $D_{adhering}$ were both increased significantly by radiation. In contrast, in adenocarcinoma-bearing preparations, $F_{rolling}$ and $D_{adhering}$ were either unchanged (in the tumour centre) or reduced (in tumour periphery and the normal tissue surrounding the tumour) by radiation. Radiation did not cause changes in haemodynamics in these preparations, thus the observed changes in leucocyte rolling and adhesion could not be accounted for by haemodynamic factors. These results indicate that: (1) in normal preparations, radiation could cause inflammation as manifested by increased leucocyte rolling and adhesion; and (2) in tumour-bearing preparations, radiation caused changes in the vascular surface properties such that they became less adhesive to leucocytes. Such differences in radiation response may have important implications for radiation therapy and provide new insights into the unique features of tumours.

It is widely known that radiation exposure causes tissue injury manifested by vascular permeability increase, inflammation, loss of blood vessels and tissue fibrosis (Baker & Krochak, 1989). The initial events after radiation which lead to these abnormalities may be twofold: release of inflammatory mediators (e.g. histamine) from mast cells (Lasser & Stenstrom, 1954) and direct damage to endothelial cells. Several features of endothelial damage have been identified. For example, it has been shown that radiation causes endothelial cells to release prostaglandins, chemoattractants and mitogenic factors (Matzner *et al.*, 1988; Eldor *et al.*, 1989; Witte *et al.*, 1989). Among other things, one important event likely to occur in the blood vessels after irradiation is an increase in interaction of leucocytes with vascular surfaces. Leucocyte adhesion to the endothelial surface can be enhanced by increased expression of P-selectin adhesion molecules as a result of histamine stimulation (Lorant *et al.*, 1991), and also by chemoattractants released by the endothelial cells. Indeed, it has been shown in an *in vitro* system that neutrophil adhesion to irradiated endothelial cells is increased (Dunn *et al.*, 1986). However, to the best of our knowledge, the effect of radiation on leucocyte adhesion to the vascular surface has not been examined in any animal model.

Leucocyte adhesion to endothelial cells is an important component of inflammation and may significantly affect the functions of microvascular circulation (Lipowsky *et al.*, 1988), yet it is only recently that this phenomenon has been studied in tumour microvessels (Ohkubo *et al.*, 1991; Wu *et al.*, 1992). Our recent study (Wu *et al.*, 1992) indicated that leucocyte rolling and adhesion in tumour blood vessels was greatly reduced compared with that in non-tumour vessels, and such reduction was due to altered properties of endothelial cells in tumours. Thus, this study suggested that blood vessels in the tumour environment might express certain unique features.

The effects of radiation on tumour tissue have been investigated largely in terms of DNA damage and mitotic cell killing. So far, there has been no study on radiation's effect on leucocyte-endothelium interactions in tumour blood vessels. In as much as such interactions may affect tumour blood flow (Lipowsky *et al.*, 1988) and oxygenation, and subsequently the outcome of radiation therapy, it is important to characterise these interactions in tumour vasculature after radiation. Furthermore, understanding how tumour blood vessels respond to radiation exposure will provide important insights into the uniqueness of tumour blood vessels.

The two purposes of the present study were, first, to verify, in an *in vivo* model, that radiation exposure would increase leucocyte rolling and adhesion and, second, to characterise the effect of irradiation on leucocyte rolling and adhesion in tumour blood vessels. Based on the results of previous studies, we hypothesised that leucocyte rolling and adhesion would be increased by irradiation in both tumour and normal blood vessels. We tested this hypothesis by measuring leucocyte rolling and adhesion in normal and tumour microvessels in a rat skinfold window chamber at 1-2 h after a 6 Gy dose of X-rays.

### Materials and methods

### *Animal preparation*

The animal model for this study was a rat dorsal skinfold window chamber with implanted tumour. This preparation allows direct visualisation of tumour vasculature. The detailed procedure for preparing this animal model has been described fully elsewhere (Papenfuss *et al.*, 1979). Briefly, an overlapping pair of circular regions of skin, 1 cm in diameter, are removed in the opposing surfaces of the dorsal flap of Fisher rats. The fascia is dissected away, leaving a single fascial plane with its associated artery-vein pair(s). The two halves of the chamber are placed on either side of the tissue window. Cover glass is placed in the centre of each chamber half to provide a barrier to infection and dehydration. The visible tissue area within the chamber is 9 mm in diameter and 180-200 μm in thickness. For tumour-bearing preparations, a 0.1 $mm^3$ piece of rat mammary adenocarcinoma

Correspondence: M.W. Dewhirst, Box 3455, Duke University Medical Center, Durham, NC 27710, USA.
Received 3 August 1993; and in revised form 6 December 1993.

(R3230Ac) is placed on one side of the implanted chamber underneath the cover glass. Preparations are used 7–10 days after surgery, when the tumours are 3–5 mm in diameter and well vascularised. Non-tumour-bearing preparations, also used 7–10 days after surgery, serve as controls. In our experiments, the rats weighed 125–130 g at the time of the surgery, and 140–150 g at the time of experimentation.

### *Irradiation of the preparations*

Rats were first anaesthetised with sodium pentobarbital (40 mg kg $^{-1}$, i.p.), and placed in lateral recumbency. Then the preparation was irradiated using 4 MeV X-rays (Clinac 4, Varian Associates). The head, limbs and body trunk were shielded by lead blocks. Radiation was delivered at 2.0 Gy min$^{-1}$ at a source-to-target distance of 80 cm. A total dose of 6 Gy was delivered. Tissue equivalent bolus was placed on both sides of the irradiated tissue in order to ensure electronic build-up.

### *Measurements of leucocyte rolling and adhesion*

The details of the method used to measure leucocyte rolling and adhesion can be found in one of our previous publications (Wu *et al.*, 1992). Only a brief description is given here.

One to two hours after the irradiation of the preparation as described above, the femoral vein of the rat was cannulated for intravenous injection of acridine orange. The rat was then placed on a temperature-controlled microscopic stage. The window chamber was positioned underneath a fluorescence microscope (Zeiss photomicroscope III) equipped with a fluorescence filter set for fluorescein. The cover glass of the window chamber was not removed, therefore the preparation was not exposed to air during experiments. Leucocytes were stained with intravenously injected 0.5% saline solution of acridine organ (Sigma, St Louis, MO, USA), which was intermittently delivered by an infusion pump (model 341B; Sage Instruments, Boston, MA, USA). The preparation was observed with a 40 × objective (NA, 0.65). The behaviour of leucocytes in each individual microvessel was recorded by a SIT camera (model C2400, Hamamatsu Photonics, Hamamatsu City, Japan) on videotape. For best visualisation of leucocytes, a small bolus of acridine orange was injected at 0.3 ml min$^{-1}$ for 5 s immediately before each recording. Each recording lasted for 1 min, during which time the preparation was continuously illuminated by a 50 W mercury lamp. In between two recordings, the mercury light was blocked to reduce tissue damage. Bright-field illumination was used during the search for the next microvessel to be recorded.

Experiments were performed on both normal and tumour-bearing rats. In normal preparations, 5–10 post-capillary venules were recorded. For tumour-bearing rats, the preparation was divided into three regions: tumour centre, tumour periphery and surrounding normal tissue. These three regions were identified based on the following characteristics of our tumour preparations. They typically have three regions of different thickness, as shown in Figure 1. The tumour centre is the thickest, while its advancing edge is slightly thicker than the surrounding normal tissue. In each region, five microvessels were recorded. In the surrounding normal tissue, post-capillary venules were selected for observation. In the tumour centre and tumour periphery, the vascular network was irregular and could not be simply divided into arterioles, capillaries and venules (Dewhirst *et al.*, 1989). To be consistent with the normal tissue, only vessels which received converging blood flow were chosen. Vessels were selected near the top surface of the preparation (maximally 100 µm depth) for best optical quality.

Leucocyte rolling and adhesion were analysed from videotapes of each experiment. For each vessel, the numbers of rolling leucocytes ($N_{\text{rolling}}$) passing a certain point in the vessel, and of adhering leucocytes ($N_{\text{adhering}}$) within a vessel segment of known length ($L$), were counted for a 30 s observation period. The length ($L$) of each vessel was chosen as long as the vessel could accommodate up to 200 µm. Vessel diameter ($D$) and the velocity of up to ten free-flowing leucocytes were also measured. A rolling leucocyte was defined as one that marginates along the vessel wall and is clearly dissociated from the bulk blood flow. An adhering leucocyte was defined as one that stays still during 30 s of observation. Two quantities were calculated to measure leucocyte rolling and adhesion. They were (i) the average flux of rolling leucocyte ($F_{\text{rolling}}$), where $F_{\text{rolling}} = N_{\text{rolling}}/(30\ \text{s})$, and (ii) the average density of adhering leucocyte ($D_{\text{adhering}}$) where $D_{\text{adhering}} = N_{\text{adhering}}/(\pi \cdot D \cdot L)$. A pseudo-shear rate ($\gamma_5$) was also calculated for each vessel as $\gamma_5 = 8V_L/D$, where $V_L$ is the mean velocity of free-flowing leucocytes.

**Figure 1** Diagram of a tumour-bearing preparation. Such a preparation is divided into tumour centre (TC), tumour periphery (TP) and normal (N) regions. **a**, *En face* image of the preparation. **b**, A cross-section of the preparation.

### *Measurement of leucocyte count and differential*

In two separate groups of experiments, the effects of irradiation and the presence of tumour on systemic leucocyte count and differential were examined. In the first group, five rats with implanted window chambers and six control rats were irradiated at 6 Gy. The five chamber-bearing rats were irradiated in identical manner as the rats used for leucocyte rolling and adhesion experiments, while the six rats without chambers had the hindlimb irradiated to a zone extending from the knee to the ankle. The size of irradiated area in the window chamber was approximately 1 cm in diameter. Given the thickness of tissue in the window chamber of 200 µm, the volume of tissue irradiated was estimated to be 16 mm$^3$. For those rats irradiated on the hindlimb, the volume of the irradiated tissue was estimated* to be more than 200 mm$^3$. Blood samples were taken from each rat before and after irradiation. These blood samples were used for leucocyte count and differential measurements.

In the second group, blood samples were taken from nine rats with adenocarcinomas implanted in the hindlimb and 11 normal rats for leucocyte count and differential measurements. The tumour diameters in the nine rats ranged from 4 to 15 mm.

### *Statistical analysis*

Data for vessels from each region (tumour centre, tumour periphery and normal tissue) of tumour-bearing rats and

*If the irradiated portion of the hindlimb is approximated as a truncated cone, then the diameters at the two ends are roughly 6 and 16 mm. The length of the shank is approximately 20 mm. The volume of this structure would be 230 mm$^3$.

from normal rats were pooled for each experimental condition (i.e. irradiated and control). Differences in vessel diameter and in free white cell velocity for each group of vessels between irradiated and control conditions were tested with the unpaired Student *t*-test. Differences in $F_{rolling}$ and $D_{adhering}$ were assessed using the general linear model. Owing to the non-normality of $F_{rolling}$ and $D_{adhering}$ data, the Poisson error term model was fit to the data. In the analysis, adjustment was made for variations in vessel shear rate ($\gamma_S$) and between-rat differences. The purpose of adjusting for $\gamma_S$ is to account for the possible effect of blood flow shear force on leucocyte–endothelium interaction. The significance of differences in $F_{rolling}$ and $D_{adhering}$ between the two conditions was ascertained by the likelihood ratio test. Estimates for differences between irradiated and control conditions (on the log scale) of both mean $F_{rolling}$ and $D_{adhering}$ were obtained. These estimates were exponentiated to give the ratio of the mean for irradiated condition to that for control condition. This analysis was carried out with the GLIM statistical package (Numerical Algorithms Group, 1987). In the graphic presentation of $F_{rolling}$ and $D_{adhering}$, the standard error of means (s.e.m.) was calculated for each parameter among vessels from the same tissue region and under the same treatment. Data are expressed as means ± s.e.m.

$F_{rolling}$ and $D_{adhering}$ were also compared between the normal vessels of tumour-bearing preparations and the normal preparations by using the same statistical analysis as outlined above.

The effect of irradiation on the systemic leucocyte count and differential was tested with a paired Student *t*-test. An unpaired Student *t*-test was used to examine the difference in leucocyte count and differential between normal and tumour-bearing rats.

A difference was regarded significant if $P < 0.05$.

### Results

A total of 23 normal rats (seven control, 16 irradiated) and 17 adenocarcinoma-bearing rats (nine control, eight irradiated) were used for the *in vivo* leucocyte–endothelium interaction experiments. There was no apparent difference in the morphological appearance of microvessels between the normal preparations and the normal tissues in the tumour-bearing preparations. Irradiation at 6 Gy did not cause changes in the size of vessels or the velocity of free-flowing leucocytes in tumour-bearing preparations, but increased both parameters in the normal preparations (Table I).

Figure 2 shows examples of video images of leucocyte adhesion in tumour and normal vessels under control and irradiated conditions. These examples show that radiation increased leucocyte adhesion in vessels of the normal preparation, but decreased it in tumour vessels. These observations are demonstrated by the following quantitative data.

Leucocyte interaction with endothelial cells was characterised by leucocyte rolling and leucocyte adhesion. The flux of leucocyte rolling, $F_{rolling}$, and the density of leucocyte adhesion on the vascular surface, $D_{adhering}$, were measured for each tissue type (tumour-bearing *vs* normal) under each condition (control *vs* irradiated). Furthermore, the data for tumour-bearing preparations were divided according to regions within the preparation. Data for $F_{rolling}$ and $D_{adhering}$ are presented in Figures 3 and 4 respectively. In tumour-bearing preparations, in the tumour periphery and the normal tissue surrounding the tumour, both $F_{rolling}$ and $D_{adhering}$ of the irradiated group decreased significantly ($P < 0.05$) compared with the control group. However, in the tumour centre, there was no significant difference in $F_{rolling}$ or $D_{adhering}$ between the control and the irradiated groups. In contrast, in the normal preparations, radiation significantly increased both $F_{rolling}$ and $D_{adhering}$ ($P < 0.05$).

To assess the magnitude of radiation-induced changes in leucocyte rolling and adhesion, the ratios of $F_{rolling}$ and $D_{adhering}$ of the irradiated rats to their counterparts of the control rats were calculated. They are presented in Table II.

A comparison of the data between tumour-bearing and normal preparations subjected to the same treatment shows that the values for both $F_{rolling}$ and $D_{adhering}$ of normal vessels were much higher in the normal preparations than in the tumour-bearing preparations ($P < 0.05$) (note that, in both Figures 3 and 4, the scales for the two preparations are different). Furthermore, radiation caused $F_{rolling}$ and $D_{adhering}$ to change in the opposite directions in these two preparations, even though the vessels in question were normal vessels.

The effects of irradiation and tumour bearing on the systemic leucocyte count and differential were studied in two separate groups of rats. In the first group, 11 rats were irradiated at 6 Gy, and blood samples were taken before and 1 h after the irradiation. There were no statistically significant differences in the leucocyte count, the percentage of polymorphonuclear leucocytes and the percentage of lymphocytes. However, the percentage of monocytes was significantly decreased by irradiation (Table III).

In the second group, the systemic leucocyte count and differential were measured from nine rats bearing adenocarcinomas and 11 normal rats. The results are presented in Table IV. There was no statistically significant difference in any of the parameters between the tumour-bearing and normal rats.

It is known that leucocyte–endothelium interaction is affected by haemodynamics within the blood vessels; a higher shear force on the vascular surface tends to reduce leucocyte rolling and adhesion (Lipowsky *et al.*, 1988; Gallik *et al.*, 1989). For this reason, we examined whether the observed differences in leucycote–endothelium interaction in response to radiation could be accounted for by changes in haemodynamics. In each blood vessel, we measured the velocity of free-flowing white cells ($V_{wbc}$). From $V_{wbc}$ and the vessel diameter, we calculated the pseudo-shear rate ($\gamma_S$), which is assumed to be proportional to the shear force acting on the vessel surface. The data for $V_{wbc}$ and $\gamma_S$, along with diameter data, in each group of preparations under each condition, are presented in Table I. Radiation did not cause a significant change in the shear rate in any group of vessels. Therefore, we concluded that the radiation-induced changes in leucocyte rolling and adhesion were not due to changes in haemodynamics. In addition, the pseudo-shear rate for normal

**Table I** Morphometric and haemodynamic parameters. All averaged data are expressed as means ± s.e.m

| Preparation | Region | Treatment | *Number of vessels* | *Diameter (μm)* | *WBC velocity (μm s⁻¹)* | *Shear rate (s⁻¹)* |
|---|---|---|---|---|---|---|
| Tumour | Tumour center | Control | 43 | 24.8 ± 1.7 | 506.2 ± 44.3 | 216.2 ± 30.2 |
| | | Irradiated | 31 | 24.1 ± 1.7 | 477.6 ± 53.9 | 187.0 ± 23.9 |
| | Tumour periphery | Control | 31 | 26.5 ± 2.5 | 363.4 ± 40.5 | 126.0 ± 15.9 |
| | | Irradiated | 19 | 25.0 ± 2.1 | 467.0 ± 71.5 | 172.7 ± 30.8 |
| | Surrounding normal | Control | 37 | 33.6 ± 3.8 | 369.4 ± 29.1 | 118.2 ± 16.5 |
| | | Irradiated | 33 | 32.0 ± 3.1 | 339.3 ± 39.5 | 111.8 ± 17.2 |
| Normal | | Control | 70 | 37.6 ± 2.4 | 410.0 ± 30.2 | 108.4 ± 12.4 |
| | | Irradiated | 145 | 44.3 ± 1.5[a] | 476.9 ± 22.8[a] | 93.2 ± 4.8 |

[a]Significantly greater than its control counterpart ($P < 0.05$).

vessels of tumour-bearing preparations was not different from that in normal preparations. Thus, the observed differences in leucocyte rolling and adhesion in normal vessels between the two types of preparations could not be accounted for by differences in haemodynamics.

### Discussion

In this investigation, we tested the hypothesis that leucocyte interactions with endothelial cells, i.e. leucocyte rolling and adhesion, would be increased by ionising radiation in the blood vessels of both tumour and normal tissues. We performed the experiments in a tumour microcirculatory preparation which allows direct visualisation of leucocyte behaviour in individual microvessels. We found that 1–2 h after exposure to 6 Gy irradiation, leucocyte rolling and adhesion significantly increased in the normal preparations. In contrast, leucocyte rolling and adhesion significantly decreased in both tumour periphery and surrounding normal blood vessels in the tumour-bearing preparations. These

**Figure 2** Digitised video images of leucocyte adhesion in microvessels. Bright white dots are fluorescently labelled leucocytes. These images were averaged from several video frames. Thus only adhering leucocytes can be visualised as round dots. **a**, Normal preparation, control condition. **b**, Normal preparation, irradiated condition. **c**, Tumour-bearing preparation, control condition. **d**, Tumour-bearing preparation, irradiated condition.

**Figure 3** Comparison of flux of rolling leucocytes between control (■) and irradiated (□) preparations. *The quantity measured after radiation exposure is significantly *lower* than under control conditions ($P<0.05$). **The quantity measured after radiation exposure is significantly *higher* than under control conditions ($P<0.05$). Note that the scales for the two preparations are different. Error bar represents s.e.m.

**Figure 4** Comparison of density of adhering leucocytes between control and irradiated preparations. *The quantity measured after radiation exposure is significantly *lower* than under control conditions ($P<0.05$). **The quantity measured after radiation exposure is significantly *higher* than under control conditions ($P<0.05$). Note that the scales for the two preparations are different. Error bar represents s.e.m.

changes in leucocyte–endothelium interactions could not be accounted for by changes in shearing forces on luminal surface of the blood vessels. Therefore, the results of this study support our hypothesis for normal preparations, but not for tumour-bearing preparations.

Increases in *in vitro* leucocyte–endothelium interactions after irradiation have been observed by other investigators. Dunn *et al.* (1986) reported that neutrophil adhesion to cultured endothelial cells was significantly increased 72 h after the endothelial cells were irradiated at a dose of 5 Gy. Matzner *et al.* (1988) reported that irradiation of endothelial cells caused release of a lipid neutrophil chemoattractant. This release of chemoattractant was rapid and radiation dose dependent. It was observed at a minimum dose of 5 Gy, and as early as 10 min after irradiation. It reached maximum at 1 h and lasted for 24 h. These results suggest that endothelial cells are capable of attracting leucocytes and probably promote leucocyte adhesion in response to radiation. In addition, in intact tissues, mast cells respond to radiation by releasing histamine, which, in turn, can up-regulate the expression of adhesion molecule P-selectin on endothelial cells (Lorant *et al.*, 1991). P-selectin is known to participate in the initial leucocyte–endothelium interaction, i.e., leucocyte rolling, and thus promoting leucocyte adhesion (Smith, 1993). Our study confirms that, in normal preparations, *in vivo* leucocyte rolling and adhesion increase 1–2 h after radiation exposure. In addition, we also observed in a related study that, at 24 h after irradiation, leucocyte rolling and adhesion in the same preparations were similar to their levels at 1–2 h (data not shown). Thus, our results are consistent with the time course of chemoattractant release reported by Matzner *et al.* (1988).

There are two surprising findings in this study. The first is that radiation exposure caused a decrease in leucocyte–endothelium interactions in the tumour-bearing preparations. The second is that the normal vessels surrounding tumour tissues had lower baseline adhesivity to leucocytes than the vessels in normal preparations, and radiation resulted in opposite reactions in these two groups of vessels. Such a contrast between tumour-bearing and normal preparations suggests that important differences exist between the tumour and normal environment. These differences affect endothelial adhesiveness not only within the tumour, but also in its vicinity. Furthermore, these differences can be amplified by radiation exposure.

**Table II** Ratios of $F_{rolling}$ and $D_{adhering}$, irradiated to control

| | *Tumour-bearing preparations* | | | |
|---|---|---|---|---|
| | *Tumour center* | *Tumour periphery* | *Normal* | *Normal preparations* |
| $F_{rolling}$ | 0.83 | 0.50[a] | 0.34[a] | 2.2[b] |
| $D_{adhering}$ | 0.91 | 0.53[a] | 0.37[a] | 1.50[b] |

[a]Significantly less than 1.0 ($P<0.05$). [b]Significantly greater than 1.0 ($P<0.05$).

Interactions between leucocytes and endothelial cells in tumour microvessels have been studied previously. Ohkubo *et al.* (1991) reported that interleukin 2 increased leucocyte adhesion to both normal and tumour microvascular endothelium in VX2 carcinoma implanted in rabbit ear chambers. In our previous studies (Dewhirst *et al.*, 1992; Wu *et al.*, 1992), we reported that inflammatory mediators such as bradykinin, bacterial lipopolysaccharide (LPS) and TNF-α could increase leucocyte rolling and adhesion in both tumour and normal blood vessels, although these increases were much higher in the normal than in the tumour vessels. It is very interesting to compare the results of these previous studies with the current study. For vessels of normal preparations, leucocyte rolling and adhesion were increased by applied inflammatory mediators in the earlier studies, and by 6 Gy radiation in this study. However, for vessels of tumour-bearing preparations, including the vessels in the normal tissue surrounding tumours, leucocyte rolling and adhesion were increased by applied inflammatory mediators in the earlier study, but were *decreased* by 6 Gy radiation in this study. Therefore, for normal preparations, radiation had similar effects as inflammation, whereas for tumour-bearing preparations, radiation had opposite effects to those observed with inflammatory mediators.

The observed radiation-related differences in leucocyte rolling and adhesion between normal and tumour preparations could not be attributed to any systemic differences in leucocyte count and differential. In two separate sets of experiments, we examined the effects of irradiation and tumour bearing on systemic white cell count and differential. In the first set, we found that irradiation of either window chambers or rat hindlimb did not cause any change in systemic leucocyte count and differential, except for a drop in monocyte percentage. The systemic effect of irradiation would be expected to be more significant for hindlimb irradiation than for window chamber irradiation since the volume of irradiated tissue was much larger for the former case. Given the fact that monocytes constitute a very small percentage of all the leucocytes, it is very unlikely that the observed drop in monocyte count after irradiation could account for the differences in leucocyte rolling and adhesion in the microvessels. In the second set, we found no statistically significant changes in systemic leucocyte count and differential between normal rats and the rats bearing adenocarcinoma on the hindlimb, which had much larger volume than the tumours implanted in the window chamber. Therefore, the observed differences in leucocyte behaviour in the microcirculatory environment between normal and tumour-bearing preparations could not be accounted for by any difference in systemic white cell parameters.

The difference in the response of blood vessels to irradiation between the two tissue types could be due to (1) differences between endothelial cells located in normal tissues and those in tumours and/or (2) differences in the parenchymal cells between normal and tumour tissues. For the first possibility, it is known that endothelial cells in the tumour environment are constantly subjected to acidic and hypoxic

**Table III** The effect of irradiation on systemic leucocyte count and differential

| | *Leucocyte count* ($\times 10^3$ $mm^{-3}$) | *Leucocyte differential (%)* *Polymorphonuclears* | *Lymphocytes* | *Monocytes*[a] |
|---|---|---|---|---|
| Preirradiation | 7.56±1.56 | 18.5±2.8 | 76.5±3.1 | 5.1±0.9 |
| Post-irradiation | 7.85±1.55 | 16.2±1.9 | 81.0±2.0 | 2.8±0.6 |
| Pre–post ratio | 1.00±0.14 | 1.35±0.23 | 0.95±0.04 | 2.32±0.59 |

[a]Significantly different between pre- and post-irradiation by the Student *t*-test ($P<0.05$).

**Table IV** The effect of tumour bearing on systemic leucocyte count and differential

| *Preparation* | *Leucocyte count* ($\times 10^3$ $mm^{-3}$) | *Leucocyte differential (%)* *Polymorphonuclears* | *Lymphocytes* | *Monocytes* |
|---|---|---|---|---|
| Normal | 8.13±1.45 | 18.2±2.1 | 76.3±2.7 | 5.6±0.9 |
| Tumour-bearing | 8.81±1.64 | 25.4±3.8 | 66.7±4.7 | 7.9±1.7 |

conditions (Vaupel *et al.*, 1989), as well as tumour-derived factors such as vascular permeability factor (also known as vascular endothelial growth factor) (Senger & Dvorak, 1992). Compared with normal endothelial cells, they are known to express certain phenotypic differences such as increased proliferation rate (Denekamp & Hobson, 1982). Several unique features of tumour blood vessels, such as increased permeability (Gerlowski & Jain, 1986; Dvorak *et al.*, 1988) and decreased leucocyte adhesion (Wu *et al.*, 1992), may be very possibly due to altered endothelial properties in tumours. Thus, it is possible that tumour endothelial cells have a different response to radiation than normal endothelial cells.

The second possibility is that the tumour parenchymal cells (tumour cells and/or other cells in tumour such as macrophages) respond to radiation by releasing mediators which are different than those released by normal cells. It is widely recognised that inflammatory mediators such as histamine are released by mast cells following radiation (Lasser & Stenstrom, 1954). Histamine can facilitate leucocyte adhesion for reasons discussed earlier. However, it is not known whether radiation could release certain substances, preferentially from tumour tissues, which can inhibit leucocyte adhesion. Such substances, if they exist, would lead to suppression of leucocyte adhesion in tumour-bearing tissues.

Of the two possibilities discussed above, the second one, i.e. mediator release from tumour parenchymal cells following radiation, seems more plausible. We found that the normal blood vessels in the vicinity of tumours had a similar response to radiation as tumour vessels. This observation suggests that tumour tissue releases certain mediators which affect the nearby normal vessels. It has been recognised that, owing to higher interstitial pressure in tumours, tumour interstitial fluid filters out of the tumour into the surrounding normal tissue (Boucher *et al.*, 1990). Tumour-derived mediators could also reach vessels of the surrounding normal tissue via blood flow. Thus it is conceivable that tumour-derived mediators could affect the surrounding normal vessels.

It is not clear at this time whether the phenomenon reported in this study is also present in other tumour types. There have been a few reports which suggest that reduced leucocyte adhesion occurs in other tumours. For example, a report by Ohkubo *et al.* (1991) indicated that, under control conditions, leucocyte adhesion to microvascular endothelium in VX2 carcinoma was lower than that in normal tissues. Groves *et al.* (1991) reported that while the expression E-selectin was present on the endothelial cells of squamous cell carcinomas, it was undetectable on vessels of basal cell carcinoma. Kuzu *et al.* (1993) reported that the expression of several important adhesion molecules for leucocyte adhesion (i.e. ICAM-1, VCAM-1, E-selectin and P-selectin) was markedly reduced in vascular tumours. Reduced expression of adhesion molecules would be expected to result in reduced leucocyte adhesion. Therefore, it is possible that reduced leucocyte adhesion is not specific to one tumour model, but rather due to certain common features shared by many tumours. Similarly, radiation-induced changes in leucocyte adhesion may not be limited to just one tumour.

If this phenomenon is also present in other tumours, it would have important implications for radiation therapy. The difference in leucocyte adhesion following radiation would lead to more leucocyte-releated tissue injury in normal tissues than tumour tissues. Since leucocyte adhesion to endothelial cells increases vascular resistance (Lipowsky *et al.*, 1988), such a difference could alter the blood flow distribution between tumour and normal tissues and affect the outcome of radiation therapy by interfering with oxygenation (Jain, 1988). Finally, adoptive immunotherapy (Rosenberg, 1991) would only succeed in tumours in which the lymphocytes used for the therapy could adhere to and trasverse the endothelial cells. If a tumour had a response to radiation similar to our observation, then it would be undesirable to combine radiation therapy with this type of immunotherapy.

In summary, we have demonstrated that blood vessels in tumour-bearing tissues became less adhesive to leucocytes after radiation, whereas those in normal tissues increased their adhesiveness to leucocytes. Such differences may lie in the differences between tumour and normal endothelial cells, and/or the differences in tissue response to radiation between tumour and non-tumour tissues.

The authors would like to thank Ms Jeri Edwards for performing the animal surgeries and Dr Edgardo Ong for the assistance in experiments. This study was supported by a grant from the NIH/NCI 1RO1CA40355.

### References

BAKER, D.G. & KROCHAK, R.J. (1989). The response of the microvascular system to radiation: a review. *Cancer Invest.*, **7**, 287–294.

BOUCHER, Y., BAXTER, L.T. & JAIN, R.K. (1990). Interstitial pressure gradients in tissue-isolated and subcutaneous tumours: implications for therapy. *Cancer Res.*, **50**, 4478–4484.

DENEKAMP, J. & HOBSON, B. (1982). Endothelial cell proliferation in experimental tumours. *Br. J. Cancer*, **46**, 711–720.

DEWHIRST, M.W., TSO, C.Y., OLIVER, R., GUSTAFASON, C.S., SECOMB, T.W. & GROSS, J.F. (1989). Morphologic and hemodynamic comparison of tumour and healing normal tissue microvasculature. *Int. J. Radiat. Oncol. Biol. Phys.*, **17**, 91–99.

DEWHIRST, M.W., VINUYA, R.Z., ONG, E.T., KLITMAN, B., ROSNER, G., SECOMB, T. & GROSS, J. (1992). Effects of bradykinin (BK) on tumour and granulating normal tissue microvascular hemodynamics. *Radia. Res.*, **130**, 345–354.

DUNN, M.M., DRAB, E.A. & RUBIN, D.B. (1986). Effects of irradiation on endothelial cell–polymorphonuclear leukocyte interactions. *J. Appl. Physiol.*, **60**, 1932–1937.

DVORAK, H.F., NAGY, J.A., DVORAK, J.T. & DVORAK, A.M. (1988). Identification and characterization of the blood vessels of solid tumours that are leaky to circulating macromolecules. *Am. J. Pathol.*, **133**, 95–109.

ELDOR, A., VLODAVSKY, I., FUKS, Z., MATZNER, Y. & RUBIN, D.B. (1989). Arachidonic metabolism and radiation toxicity in cultures of vascular endothelial cells. *Prostaglandins Leukotrienes & Essential Fatty Acids*, **36**, 251–258.

GALLIK, S., USAMI, S., JAN, K.-M. & CHIEN, S. (1989). Shear stress-induced detachment of human polymorphonuclear leukocytes from endothelial cell monolayers. *Biorheology*, **26**, 823–834.

GERLOWSKI, L.E. & JAIN, R.K. (1986). Microvascular permeability of normal and neoplastic tissues. *Microvasc. Res.*, **31**, 288–305.

GROVES, R.W., ALLEN, M.H., BARKER, J., HASKARD, D.O. & MACDONALD, D.M. (1991). Endothelial leucocyte adhesion molecule-1 (ELAM-1) expression in cutaneous inflammation. *Br. J. Dermatol.*, **124**, 117–123.

JAIN, R.K. (1988). Determinants of tumour blood flow: a review. *Cancer Res.*, **48**, 2641–2658.

KUZU, I., BICKNELL, R., FLETCHER, C.D. & GATTER, K.C. (1993). Expression of adhesion molecules on the endothelium cf normal tissue vessels and vascular tumors. *Lab. Invest.*, **69**, 322–328.

LASSER, E.C. & STENSTROM, K.W. (1954). Elevation of circulating blood histamine in patients undergoing deep roentgen therapy. *Am. J. Roentgenol.*, **72**, 985–988.

LIPOWSKY, H.H., HOUSE, S.D. & FIRRELL, J.C. (1988). Leukocyte endothelium adhesion and microvascular hemodynamics. *Adv. Exp. Med. Biol.*, **242**, 85–93.

LORANT, D.E., PATEL, K.D., MCINTYRE, T.M., MCEVER, R.P., PRESCOTT, S.M. & ZIMMERMAN, G.A. (1991). Coexpression of GMP-140 and PAF by endothelium stimulated by histamine or thrombin: a juxtacrine system for adhesion and activation of neurophils. *J. Cell Biology*, **115**, 223–234.

MATZNER, Y., COHN, M., HYAM, E., RAZIN, E., FUKS, Z., BUCHANAN, M.R., HAAS, T.A., VLODAVSKY, I. & ELDOR, A. (1988). Generation of lipid neutrophil chemoattractant by irradiated bovine aortic endothelial cells. *J. Immunol.*, **140**, 2681–2685.

NUMERICAL ALGORITHMS GROUP (1987). *The GLIM System Release 3.77 Manual*. 2nd edn. Numerical Algorithms: Oxford.

OHKUBO, C., BIGOS, D. & JAIN, R.K. (1991). Interleukin 2 induced leukocyte adhesion to the normal and tumour microvascular endothelium *in vivo* and its inhibition by dextran sulfate: implications for vascular leak syndrome. *Cancer Res.*, **51**, 1561–1563.

PAPENFUSS, D., GROSS, J.F., INTAGLIETTA, M. & TREESE, F.A. (1979). A transparent access chamber for the rat dorsal skin fold. *Microvasc. Res.*, **18**, 311–318.

ROSENBERG, S.A. (1991). Immunotherapy and gene therapy of cancer. *Cancer Res.*, **51** (Suppl.), 5074s–5079s.

SENGER, D.R. & DVORAK, H.F. (1992). Vascular permeability factor: a protein mediator that induces tumour vessel hyperpermeability and promotes endothelial cell growth. In: *Human Astrocytomas*, Black, P.McL. & Lampson, L. (eds). Blackwell Scientific Publications: Oxford.

SMITH, C.W. (1993). Endothelial adhesion molecules and their role in inflammation. *Can. J. Physiol. Pharmacol.*, **71**, 76–87.

VAUPEL, P., KALLINOWSKI, F. & OKUNIEFF, P. (1989). Blood flow, oxygen, nutrient supply and metabolic microenvironment of human tumours: a review. *Cancer Res.*, **49**, 6449–6465.

WITTE, L., FUKS, Z., HAIMOVITZ-FRIEDMAN, A., VLODAVSKY, I., GOODMAN, D.S. & ELDOR, A. (1989). Effects of irradiation on the release of growth factors from cultured bovine, porcine, and human endothelial cells. *Cancer Res.*, **49**, 5066–5072.

WU, N.Z., KLITZMAN, B., DODGE, R. & DEWHIRST, M.W. (1992). Diminished leukocyte–endothelium interaction in tumour microvessels. *Cancer Res.*, **52**, 4265–4268.